# The Medical Mind and Conscience

**Published:** 1889-09-14

**Authors:** 


### The Doctor's Pulpit.

### THE MEDICAL MIND AND CONSCIENCE.

There are metaphysicians who affirm that the conscience cannot be educated. The understanding or mind can be instructed, but not the conscience. The conscience can be made more sensitive or less sensitive, more imperious or less, by training, but it cannot be enlightened or made gnorant. That is a metaphysical question into which we need not enter. For practical purposes every calling has its rules and standards of conduct, and so also has the medical profession.

Medicine has no written bible or "book of faith" comparable to the New Testament of the Christians, the Old Testament of the Hebrews, or the Koran of the Moslems. It has, however, a faith of its own contained in the "traditions of the elders," and handed down from generation to generation in a more or less modified form. It is wonderful what a large proportion of the members of the profession are "obedient to the faith." Probably not even the clergy themselves show more respect for the Prayer-book and other standards of doctrine, ceremonial, and life, than do the physicians and surgeons of the world for their unwritten traditions. There is a certain merit in this decided conservatism. Old convictions and customs have earned the right to respectful treatment. It is true that what is old is often feeble; and that which becomes feeble must in the very nature of things be removed. But Nature, for the most part, removes what is old in a very gentle fashion, and human beings may well take a hint from her in this regard.

Many people have a great contempt for what they call "medical etiquette." It is much to be wished that a less contemptible name than "etiquette" had been used for the thing signified, but the thing itself, in the hands of right-minded persons, is eminently worthy. Medical etiquette, or, as it is now more commonly called in professional circles, "medical ethics," is a subject not only well worthy of the study of all medical students, but one which must be studied if they are to be honourable and successful practitioners. One branch of medical ethics may very well be discussed under the heading of the "medical mind and conscience."

Is there any practical difference between the medical and any other mind and conscience? Without affirming that there is, it is at least true to say that the medical mind of the present day has a distinct individuality of its own. When a man has passed the student stage and settled down into practice, it is surprising how quickly and willingly he assumes a frockcoat, and with it the distinguishing outward marks and inward characteristics of "the doctor." By the time he has been twenty years in his profession, nineteen strangers in every twenty who have never seen him before will make very successful guesses at his calling and status. What is the most striking characteristic of the modern medical mind? Is it not that it sets the greatest possible value on material facts? That which can be touched with the finger, seen by the naked eye or the highest power of the microscope, heard by the stethescope, registered by the thermometer, measured by other instruments of precision, or acted upon by drugs—that to the medical mind is tangible, real, indisputable; and anyone who approaches the average doctor charged with facts of that kind, be they old or be they new, is sure of immediate attention. In the region of demonstrable facts the medical man is the staunchest conservative in the world, and at the same time the most progressive radical. He will stick to any doctrine however antiquated, provided it is based on material facts; he will accept any theory however new and strange if sufficient material facts can be brought to prop it up. He is the very opposite of the Frenchman who, when told that the facts were against him, exclaimed, with his national shrug, "So much the worse for the facts." The doctor, on the other hand, has a reverence for indisputable facts which is both comical and pathetic as well as honourable.

Along with this reverence and submission to fact there is in the modern doctor an equally remarkable timidity and distrust of mere theorising and dialectic. The medical man of to-day hates and fears wide-ranging speculation as much as his predecessors loved it. Even drugs whose uses have been proved by scores of years of experience in the hospital and the family, he submits with anxious scepticism to the much less reliable tests of the physiological laboratory. The modern doctor is too often timid to a fault. He is dominated by the physiological experimentalist. He is, in many instances, feeble-minded even to mental suicide. He annihilates his own personality in order that he may worship undisturbed the fetish of prominent names. This is fatal to skilled and successful practice, as it is absolutely destructive of manhood. There is, besides, not the least necessity or justification for it. The average medical student is in these times so intelligently grounded in the science and art of his professional work, that honest study and conscientious practice cannot fail to make him a competent and self-reliant practitioner in a few years; and that whether his work lie in the country village or in a metropolitan hospital. It has been the fortune of the writer to see the practice of some of the most eminent as well as of some of the least-known members of the medical profession. He has no hesitation in saying that in all ordinary work the conscientious and intelligent village practitioner of modern days, is quite the equal of the conscientious and intelligent hospital physician. Of course, in certain departments of practice the pure specialist has no rival. But the ordinary diseases of mankind, which fall to the lot of the ordinary doctor to treat, require neither specialist nor consultant if the family practitioner be an honest and intelligent man. The timidity of many doctors, and the excessive respect they show for great names, is as unjustifiable in itself as it is injurious both to the doctor and his patients.

In addition to excessive mental timidity, and a conscience sensitive mainly to material facts, the modern doctor is handicapped by his deficient general education and comparative ignorance of literature. Many medical men do not even know that medical books are for the most part not literature at all, but mere business manuals, exactly the same as books on carpentery or arithmetic. And yet many of them feed their minds exclusively on such books; and thus become not men whose calling it is to be doctors, but mere doctors who think they have no calling to be men.

The result of all this is vastly injurious. It puts doctors as a body out of sympathy with common affairs; makes them as ignorant and as helpless as children in public business; exposes them to the ridicule of experienced men of the world ; and constitutes them a class apart, who, though sought after for their special services, are often regarded with a kind of amused pity as pedants and unpractical obstructives.

The appreciation of material facts which is so characteristic of the mental attitude of the modern doctor, and the sensitiveness of his conscience within this limited range, are altogether admirable so far as they go, and well deserve consideration and adoption by other classes. But they do not go far enough. They make many doctors one-eyed, one-armed, and limping specialists instead of full men. "Reading maketh a full man," said Bacon, but he would have regarded with amazement any reader who should propose to make himself a "full man" on an exclusive diet of medical books. The doctor needs to be balanced by the man; the shrivelled skin of the specialist must be filled out by the springing sap and the robust energy of a liberal manhood ; professional ethics must be verified and expanded by human sympathies ; the pedant must be crucified that the man may live. Never before had medical men so rare an opportunity of making themselves the trusted scientific guides and earthly friends of their fellow-men as now. If they take a purely selfish and professional view of their duties that opportunity will pass away, and doctors will fall back to the despised position they occupied two hundred years ago. If they interpret their special functions liberally, and resolve to be men as well as doctors, nothing can prevent them reaching the very highest place in the esteem of their worthiest contemporaries.

